# Positive Identity as a Positive Youth Development Construct: A Conceptual Review

**DOI:** 10.1100/2012/529691

**Published:** 2012-04-30

**Authors:** Sandra K. M. Tsang, Eadaoin K. P. Hui, Bella C. M. Law

**Affiliations:** ^1^Department of Social Work and Social Administration, Faculty of Social Sciences, The University of Hong Kong, Pokfulam Road, Hong Kong; ^2^Division of Learning Development and Diversity, Faculty of Education, The University of Hong Kong, Pokfulam Road, Hong Kong

## Abstract

Identity is a core construct in psychology because it refers to how a person addresses issues dealing with who that person is. Important theorists studying the concept of identity, like Erikson, Marcia, and Higgins, assert that identity is organized,is learned, and is dynamic, and a subjective evaluation of an individual's identity has emotional consequences for that individual. Adolescents who can cultivate a clear and positive identity after their developmental struggles during adolescence often advance more smoothly into adulthood. This paper reviews literature on the nature and structure of identity and examines its importance on adolescent developmental outcomes. It traces significant determinants of identity and proposes strategies for cultivation of positive identity. Observations on current research gaps in the study of identity and future research directions will also be discussed.

## 1. Background

Within the field of psychology, the nature and development of identity and related concepts like self and self-identity have attracted voluminous research over many decades. Studies began with Freud's early writings and were popularized by Erikson's theoretical expositions. Since the 1960s, Marcia's empirical operationalization of the concept has led other contemporary theorists like Higgins [1], Berzonsky et al. [2], Grotevant [3] to develop it further. According to the PsycInfo database, in the 20 years from 1985 to 2004, there were over 72,000 studies on the topic. Review of such literature suggests that identity is an important social science concept. There are structural and process components in identity. Structurally, identity can be meaningfully organized into general, physical, psychological, social, and spiritual domains. Identity formation also involves dynamic processes as identity evolves along with persons development throughout their life span. Identity is amenable to extrapersonal influences like environmental changes and life experiences as well as intrapersonal identity processes, including exploration, commitment, and reconsideration. There is also evidence that gender, age, and culture patterns at different times affect the development of identity.

Adolescence is a developmental stage characterized by rapid and extensive physical and psychosocial changes which often present developmental crises that challenge the adolescent's coping ability. Successful coping culminates in the formation of a clear and positive identity that can facilitate future development and productive use of personal resources. Problematic coping might make the person vulnerable to emotional and behavioral problems. How adolescents address what they experience in adolescence to formulate their identity has a pivotal impact on their subsequent life journeys.

## 2. Definition of Identity

Identity basically refers to how a person answers the question “Who am I?” S. Sharma and M. Sharma [4] (page 119) said “Identity is an umbrella term used throughout the social sciences to describe an individual's comprehension of him or herself as a discrete, separate entity”. Psychologists most commonly use the term “identity” to describe “personal identity” or the idiosyncratic things that make a person unique. For example, Grotevant [3] (page 1119) defined identity as the “distinctive combination of personality characteristics and social style by which one defines oneself and by which one is recognized by others”. Cognitive psychologists tend to focus on the awareness of self and the capacity for self-regulation, while sociologists examine social identity and role behavior.

Erikson [5] and subsequent researchers (e.g., Ashmore and Jussim [6]; Misra [7]; Thoits [8]) conceptualized identity as individual versus societal level phenomena. At the individual level, they differentiated amongst “I” (self/identity as knower/subject/process), “Me” (self as known/object/structure), and “Self” (“the self”, “selves/identities” and “self-esteem”) [8]. At the societal level, Misra [7] emphasized the important role of culture in constructing the structure and processes of self. Others (e.g., Hardie et al. [9]; Thoits and Virshup [10]) also tried to explore the connections between personal identities (similar to self-concept), role/relational identities (definition of self in a particular role in an interactional context), and social/collective identities (identification of self with a social group, culture or category).

Identities have also been conceptualized as blocks that build up the global, unified self-concept that enables a person to function with coherence [11]. The development of clear and positive identity/identities involves building self-esteem, facilitating exploration of and commitment to self-definition, reducing self-discrepancies, and fostering role formation and achievement.

## 3. Theories on Identity

Following the individual and social concepts of identity, there are theories addressing these two levels, as well as theories defining the structure and dynamics of identity development.

### 3.1. Erikson's Identity Theory

Erikson [5] is the most prominent theorist to draw attention to the nature and development of identity. He proposed an eight-stage progression of human development over the human life span: infancy, early childhood, childhood, puberty, adolescence, early adulthood, middle adulthood, and late adulthood. Each stage is marked by a psychosocial crisis that involves confronting a fundamental question. The stages are described in terms of alternative traits that are potential outcomes from each crisis. Development is enhanced when a crisis is resolved in favour of the healthy alternative. In the adolescence stage, an identity crisis occurs when adolescents seriously question their essential personal characteristics, their views of themselves, their concerns about how others view them, or their doubts about the meaning and purpose of their existence. The outcome could be either healthy and coherent identity formation or identity confusion. He also postulated that identity can be examined at the ego, personal, and social levels. 

Erikson's theory established a foundation for research on ego, identity development, and intervention models. Since then, studies on identity have expanded to include a consideration of individual differences; the search for, discovery of, and utilization of innate potentials; critical problem-solving skills; social responsibility; integrity of character; the impact of social and cultural contexts on identity formation and development [12].

### 3.2. Marcia's Identity Status Theory

Marcia [13] and Marcia et al. [14] popularized Erikson's construct of identity by proposing four identity statuses (identity diffusion, foreclosure, moratorium, and identity achievement) as possible outcomes of identity formation. She also proposed two dimensions, namely, exploration and commitment, which influence identity formation. Exploration refers to a process of actively questioning and searching for adult roles and values in the various domains of adolescent life. Commitment refers to firm decisions regarding aspects, such as vocation, political ideology, religion, and social roles, and includes specific strategies for achieving personal goals and a desired life path. Identity diffusion is a status in which exploration has not occurred nor has any commitment been made. Foreclosure denotes the status when commitment has been made but is not supported by adequate exploration. Moratorium refers to an active exploration of identity with weak commitment, possibly trying on several different masks at the same time. Identity achievement is the status in which the individual has explored his or her identity potential fruitfully and can now commit to a particular identity. Diffusion is often considered the least adaptive status [15]. 

Subsequent research [16, 17] not only provided strong support for the four-status model but also allowed exploration to be separated into two types: in-depth exploration and reconsideration. In-depth exploration refers to the extent to which adolescents actively assess the merits of their current choices of commitment, either by themselves or in consultation with significant others. Reconsideration of commitment refers to comparing present commitments with possible alternative commitments. In-depth exploration was found to correlate positively with agreeableness, conscientiousness, and openness to experience [16]. However, it may also contribute to negative consequences such as having an unclear self-concept, coupled with emotional instability. Reconsideration evokes short-term costs as it creates uncertainties and holds up progress, and adolescents stuck in this process might exhibit emotional or behavioral problem; but reconsideration should have a long-term positive effect as global contexts are changing rapidly and constant adjustment should be an essential strategy for achievement, or even survival. An example cited by Crocetti et al., [16] involved an Italian college freshman who left his home town for study. He expressed the need to find another best friend in his current context even though he had a very good friend in his home town because he needed social support nearby. This freshman is committed to his existing friendship but has new needs or even anxieties arising from his new environment and is thus reconsidering strategic alternatives in order to optimize his condition. Examples like this one clearly illustrate that this additional concept of reconsideration has enriched the identity formation model by taking it from being more linear to being more cyclical.

### 3.3. Higgins's Self-Discrepancy Theory

Instead of centering on the individual, Higgins' self-discrepancy theory [1] focuses on the discrepancies between three self-domains and two standpoints on the self. The three self-domains are (i) actual self—how a person or others see the attributes that the person possesses; (ii) ideal self—the attributes that a person or others would like the person to possess; (iii) ought self—the attributes that the person or others think the person should possess. The two standpoints on the self are (i) a person's own and (ii) significant others' (such as parents and teachers) sets of attitudes or values that can be judged. The self-domains and the standpoints on the self can be combined and used to generate six self-state representations: actual/own, actual/other, ideal/own, ideal/other, ought/own, and ought/other. The nature and the scale of the discrepancies between the different types of self-states affect the person's emotional vulnerabilities [18]. For example, discrepancies between the actual/own and ideal/own self-states are associated with disappointment and dissatisfaction; discrepancies between the actual/own and ideal/other self-states are associated with shame, embarrassment, or feeling downcast; discrepancies between actual/own and ought/other self-states are associated with fear and feeling threatened; discrepancies between actual/own and ought/own self-states are associated with feelings of moral worthlessness or weakness. However, some discrepancy is inevitable during the search for and the development of identity, and Higgins has proposed strategies to relieve the painful experiences arising from such struggles, such as changing one's actual/own self-concept or self-guides so that they are less discrepant from one another, or changing the accessibility of the discrepancies [1].

### 3.4. Theory of Possible Selves

The youngest and perhaps most promising member of the so-called Self Family in psychological studies aimed at understanding people's behavior is the notion of possible selves. Possible selves represent those elements of the self-concept that individuals could become, would like to become, or are afraid of becoming [19]. The latter is also known as Fear Possible Selves. Empirical research has supported the idea that Possible Selves could be self-regulatory and can serve as road maps for one's behavior. Oyserman et al. [20] reported studies linking increased numbers of positive Possible Selves with a reduced risk of substance use and sexual activity. In addition, Sun and Shek [21] showed that adolescents are less likely to engage in problem behavior when they have a sense of purpose and meaning in life. Exploring Possible Selves among youths in Hong Kong provides researchers, practitioners, and policy planners with a helpful way to understand our young people and sheds light on the development of prevention and intervention programmes that target youths who may be or are affected by drug use and other at risk behaviors.

### 3.5. Identity Style Theory

The increasing focus on social-cognitive variables generated much interest in studying the role that identity processing styles play in adolescent identity generation. Benzonsky's Identity Style Model postulates that three processing styles can be depicted in adolescent identity formation [22, 23]. The informational style involves the active searching and evaluation of identity-relevant information. The normative style refers to the adherence to conventions, and dependence on the expectations and feedback of significant others when confronted with identity issues. Last but not least, the diffuse-avoidant style denotes a tendency to procrastination in the handling of identity issues. Individual cognitive, social, and psychological variables like personality have been found to have an influence on such identity styles [23, 24], and the studies have now expanded to examining how significant others like family members [25], teachers, and peers affect the establishment and maintenance of such styles.

## 4. Assessment of Identity

There are at least five aspects of assessing identity: levels and domains of identity, identity statuses, identity dimensions, identity styles, and progressive developmental shifts in identity during its development.

### 4.1. Levels and Domains of Identity

Identity can be assessed on both a personal level and a social level. Global identity is conceived to be made up of identity in different domains such as physical appearance, athletic competence, scholastic competence, social acceptance, behavioral conduct, and global self-worth [26]. Different scales, including culture-specific ones, have been developed to measure specific and global identity. For example, Cheng and Watkins [27] constructed the Chinese Adolescent Self-Esteem Scales (CASES) to assess the self-esteem of Chinese adolescents in terms of seven aspects: social, academic, appearance, moral, family, physical/sport, and general self-esteem.

### 4.2. Identity Statuses

According to Meeus [17], identity development can end up in one of four possible identity statuses: diffusion, foreclosure, moratorium, and identity achievement. Each of these statuses can also be examined in connection with other psychosocial outcome correlates, such as psychological well-being, quality of life, identity congruity or discrepancies, strategies and skills in problem-solving and coping, and self-regulation abilities. Quantitative and qualitative methods (like the Identity Status interview) have been devised to assess such statuses [28].

### 4.3. Identity Dimensions

In line with the two-dimensional model of identity formation, the Utrecht-Groningen Identity Development Scale (U-GIDS) [17] includes two subscales to assess the two identity dimensions: commitment and in-depth exploration. With emerging evidence of a three-dimensional model of identity, a new exploration scale, reconsideration, was added to form the Utrecht-Management of Identity Commitments Scale (U-MICS) [16].

### 4.4. Identity Styles

Based on the Identity Style Model, Berzonsky developed and fine-tuned the Identity Style Inventory, Revised (ISI3) [29], which used 5-point Likert-type items to measure the informational, normative, and diffuse styles. The scales include 11, 9, and 10 items, respectively, and their internal consistency was around  .70.

### 4.5. Developmental Shifts in Identity

Active attempts were made to capture the dynamic evolutions in identity formation, development, and possible shifts between different identity statuses. Klimstra et al. [30] conducted a five-wave longitudinal study of 923 early-to-middle adolescents and 390 middle-to-late adolescents that provides a comprehensive understanding of change and stability in identity formation in adolescence. Three types of change and stability (mean-level change, rank-order stability, and profile stability) were assessed according to the three-dimension model of identity (commitment, in-depth exploration, and reconsideration). The study is robust in its large sample size, elaborate research design, psychometric specificity the strength of the measures used, and its sharpness in addressing theoretical postulations. The study has opened up new frontiers in identity assessment and research. It has revealed that there are changes in identity dimensions towards maturity and there is a progressive change in the way that adolescents deal with commitments.

## 5. Antecedents/Determinants of Adolescent Identity

The development of identity is a life-long process, and people at different stages of life have different identities. Stage theories of psychosocial development (e.g., Erikson's psychosocial development theory of 1968 [5], Freud's psychosexual development theory in the 1950s [31]) emphasized that human development proceeds along stages that follow the same sequential order in all people. Each stage is identified by specific developmental characteristics and tasks. Successful achievement of the task in a stage (e.g., feeling securely taken care of during the most vulnerable infancy stage; achieving a clear identity during the identity-searching adolescent stage) will provide adaptive foundational resources for progression into, and successful achievement of, the tasks in the following stages. Conversely, having stumbled in an early stage predicts fewer resources and less chance for success in dealing with the tasks encountered in subsequent stages (e.g., suffering deprivation or abuse in the dependent early childhood years will make a person untrusting of others and difficult to get along with in adulthood). Identity development is particularly vigorous in adolescence [32, 33] and the resultant identity status naturally lays the foundation for adulthood development.

In identity development, individual factors such as age, gender, physical health and appearance, intelligence, and social skills all cast significant influence on a person's real and perceived identity. Healthy, good looking, intelligent, and sociable children can more readily engage support to help them excel in actual performance, self-esteem, and even social attractiveness. Regarding the impact of age and maturity on identity, the large-scale five-wave longitudinal study of changes in adolescent identity formation by Klimstra et al. [30] found that older adolescents who become more mature with age show decreases in reconsideration, increases in in-depth exploration, and increasingly stable identity dimension profiles. How adolescents deal with commitments has more impact on identity formation than the actual changes in the commitments themselves. Grotevant [3] found that teenagers have more conflicts with their parents in early adolescence and fewer conflicts as they grow into late adolescence because both parents and adolescents understand each other better when the adolescents have a clearer identity.

Studies on gender differences consistently show that in early adolescence girls are normally more mature than boys with respect to identity formation, while boys catch up by late adolescence [30]. Gilligan [34, 35] pointed out that females define themselves in terms of relationships with other people while males define themselves through achievements [36]. Kling et al. [37] found that males had higher self-esteem than females, and that the peak difference was at the ages of 15 to 18. In Hong Kong, Watkins et al. [38] also found older adolescent girls tend to report significantly lower self-esteem than younger girls and older boys, especially in the areas of physical abilities, reading, mathematics, and general self-concept.

Aside from examining the stages of development and individual characteristics, the Social Development Theory also draws attention to the importance of the risk and protective factors of individual's significant others in the individual's identity development [39]. There are risk and protective factors in each type of significant others, specifically in the family, school, peers, community, and the internet in contemporary societies where global information and cyber relationships can be easily accessed. Surveys on Chinese families with adolescents in Hong Kong [40, 41] involving samples of over 1,000 adolescent respondents have found that fathers have a stronger influence on the development of their children's self-esteem than mothers, especially in the case of daughters. In Youngblade, Theokas, Schulenberg, Curry, Huang, and Novak's large-scale study on the risk and promotive factors of positive youth development in schools, families, and communities, it was found that each of these sectors harbor some characteristics conducive to positive youth development [42]. Affective and abundant family communication, clear rules about watching television and a healthy parental role model produced healthy youth behavior. Family conflict and aggression posed the risk of causing identity confusion and behavior problems in young people [43]. School and community safety were related to higher social competence and decreased externalizing behavior. School violence resulted in a risk of more internalizing and externalizing behavior, poorer academic performance, and lower self-esteem. In their attempt to explore the roles personality, identity styles, and family functioning play in influencing youth identity, Dunkel et al. [25] found that it was personality, instead of family functioning, that accounted more for significant variations in informational and diffuse-avoidant identity style scores. For normative identity style scores, both personality and family functioning were influential.

Regarding cultural determinants of identity, patriarchal societies like most Asian countries still show a more obvious preference for boys, while also harboring higher expectations for boys to achieve, not just for individual merits but also for collective esteem. Spencer-Rodgers et al. [44] found that Chinese and Asian Americans exhibit greater ambivalence towards self-evaluation than do Chinese and Asians from synthesis-oriented cultures. They concluded that identity development varies between Eastern collectivistic cultures and Western individualistic ones. However, with increased globalization and population movement [45], the growth of racial minorities in different societies [16], and handy connections through the internet [46], the continuing validity of this East-West collective-individualistic dichotomy will need to be evaluated. In addition, with increased attention being paid to multiple intelligences instead of mainly academic achievements, cultural expectations and their impact on identity formation and success become much more complicated.

## 6. Adolescent Identity and Developmental Outcomes

Meeus has proposed four possible identity outcome statuses, including diffusion, foreclosure, moratorium, and identity achievement, with the last being the healthiest of the four statuses [17]. Specifically, an individual's identity profile (strengths and weaknesses across the different identity domains), identity status (including the interplay of exploration and commitment, the magnitude of discrepancies between real and ideal self), and the ability of that individual to effect necessary improvement to his or her identity development, all have an impact on the immediate positive and negative aspects of the individual's well-being. In addition, it will affect one's long-term development into adulthood and the future stages of one's life span. Positive indicators include self-esteem, life satisfaction, positive effect, quality of life, environmental mastery, positive relations with others like parents [2], teachers, and peers, and self-acceptance. Negative indicators include internalizing pathology like stress, depression, and anxiety, as well as externalizing pathological behavior like hostility, aggression, loss of control, and disruptive behaviour [4, 45]. Many such indicators are included as positive youth development constructs in Project PATHS, a positive youth development program developed and validated for Chinese adolescents [47].

## 7. Promotion of the Development of Positive Identity

The enhancement of positive identity development in young people can be achieved at both the individual and the social levels. Catalano, a researcher on positive youth development programs, conceptualized positive youth identity as “the internal organization of a coherent sense of self” [48] (page 106). He found that “positive identity” was treated as a core construct in nine out of 25 effective positive youth development programs. Specific strategies adopted by these programs to enhance positive identity include the following.

### 7.1. Promoting Self-Esteem

According to Harter [49], one's evaluation of oneself, often called self-esteem, can influence identity formation and the emotions and performance related to it. Positive self-evaluation typically energizes a person while negative self-evaluation, especially when it is prolonged and hinges upon attributes that cannot be easily changed or acquired, can disturb person's emotions and performance.

Borba's Esteem Builders curriculum is one of the most comprehensive and widely used skills-based curricula [50]. Its theoretical framework is inspired by Branden [51] (page 27) who defined self-esteem as “the disposition to experience oneself as competent to cope with the basic challenges of life and as worthy of happiness.” Borba emphasized five acquired components of authentic self-esteem: (a) security, the feeling of strong assuredness; (b) selfhood, the feeling of self-worth and accurate identity; (c) affiliation, the feeling of belonging and social acceptance; (d) mission, the feeling of purpose; and (e) competence, the feeling of self-empowerment and efficacy. In Hong Kong, the Tung Wah Group of Hospitals indigenized Borba's Esteem Builder curriculum in 1993 and developed programs for use in schools from preschools to secondary schools [52, 53]. The Project PATHS developed to enhance positive youth development also built on Borba's model by working on the key components of self-esteem enhancement and identity exploration. The aim is to enhance junior secondary school students' skills in recognizing their self-image, reducing self-discrepancies, and increasing positive self-talk. The curriculum will also use societal expectations of appropriate gender roles and identity to sharpen gender-sensitive discussions. Skills taught include positive self-evaluation, assertiveness affirmation skills, and understanding and dealing with social expectations and undue negative comments.

### 7.2. Fostering Exploration and Commitment

According to Marcia's identity status theory [13], adolescents have to decide upon their own roles through experiences that expose them to opportunities and situations that challenge how they understand and manage such experiences. Their struggles and exploration through this exposure will promote a more in-depth and multiangled appraisal of their experience, build up their stress-coping abilities, and advance their problem-solving efficiency and effectiveness. Reconsiderations in the light of new circumstances can also help to refine and clarify their identity. Clarity of identity through such exploration creates the platform for identity commitment [54] and will help the adolescents build the maturity and competence needed to master further life transitions [32, 55].

### 7.3. Reducing Self-Discrepancies

During the process of identity search, adolescents often encounter discrepancies between their ideal self, real self, self-perceived self, and their self as perceived by others, or discrepancies between personal and social identities. Such discrepancies will expose adolescents to increased psychosocial risks like emotional and behavioral problems. By identifying the nature and magnitude of such discrepancies, steps can be taken to reduce these disturbing discrepancies, reinforce identity clarity and commitment, and even promote self-esteem [1, 32].

Aside from working directly on the individuals themselves, effective management of risk and protective contextual determinants is also important for fostering positive identity. Traditionally, schools and families are the two most influential developmental contexts for adolescents who normally live at home and study in schools. The physical and psychosocial environments at home and school, the resources of those entities, the opportunities they provide, the support and recognition they give to the youths, together with their rules and values all influence the identity development of the youths. Schools and families exist in specific cultural and subcultural contexts, and the characteristics of such, like gender role expectations, religiosity, and achievement expectations, also release positive or negative energies that influence identity development. In recent decades, penetration of adolescents' daily lives by the media and the internet has enabled young people to access local and global, as well as factual and virtual contexts. In urbanized cities like Hong Kong, young people can command greater information technology than their parents and even their teachers, making it very difficult for adults to provide appropriate guidance to the young [56]. The virtual environment enables the manipulation of made-up identities for explorative social interactions. How to keep such “exploration” within functional and adaptive limits is certainly a challenge to information-technology-driven modern life styles. An effective use of such channels should be able to prepare adolescents better to go through their adolescence and be best prepared for adulthood.

## 8. Research Gaps and Future Research Directions

Since the inception of the concept of identity by fore-runners in psychology, the concept has attracted numerous studies along the following themes.

1. The nature and structure of identity, and how to achieve accurate description and assessment.
2. Identity formation, including understanding the nature and magnitude of changes in the identity statuses, as well as changes in separate identity dimensions [30].
3. Connections between identity, identity dimensions, identity statuses and possible selves, and health, success, and well-being.
4. Identity management through self, and significant others, and risk and protective contexts

In terms of research methodology, there were formerly limitations to the sample sizes, the duration of research projects, and data collection methods. In recent years, there has been an expansion from cross-sectional self-report paper-and-pencil quantitative studies to the inclusion of multi-wave longitudinal studies with multimethod data collection in order to test models on how, when, and why progressive identity changes take place (e.g., Klimstra et al. [30]). In addition to studies on individual identity, there are also an increasing number of studies on social identity, and useful new themes include (a) symptoms appraisal, (b) health-related norms and behavior, (c) social support, (d) coping, and (e) clinical outcomes [4, 57]. These help to fill gaps in the research subject areas. There is increased attention given to age, gender, and cultural influences. Instead of singling out adolescence as “the” most critical stage in identity development in a life span, there is also an expanded interest in studying emerging adulthood (the lives of people from their late teens to their mid-to-late 20s in industrialized societies) [58, 59].

In view of the increasing geographical and internet-based globalization [46], pressures in changing education systems [60], economical conditions, and vocational prospects for young people, it is important that future identity studies be anchored even more firmly on specific reality situations. Examples include examining whether or not internet surfing has really created barrier-free access for people with physical disabilities; how people who use fake identities on the internet to create their social networks benefit or suffer from such fluid and real/unreal identities; and identity evolution where people live and work in different places and encounter diverse ethnicities and cultures.

## 9. Conclusion

Adolescents are the future masters of society. A clear and well-developed identity and favorable self-esteem promise positive development throughout adolescence and even across a whole life span. As identity is organized, complex, dynamic, and amenable to social influences, it is important to incorporate significant others in the adolescents' ecology in order to provide effective exposure and learning activities and to provide the support necessary [60] for helping adolescents develop a healthy identity.
